# Inhibition of P2X7 receptor in satellite glial cells contributes to electroacupuncture's analgesia in rats with CFA-induced inflammatory pain

**DOI:** 10.1186/s13020-025-01227-6

**Published:** 2025-10-05

**Authors:** Yuxin Wu, Minghui Wu, Shuxin Tian, Zhengyi Lyu, Bei Zhao, Boxi Zheng, Junying Du, Junfan Fang, Xiaofen He, Boyi Liu, Xiaomei Shao, Jianqiao Fang, Yi Liang

**Affiliations:** Department of Neurobiology and Acupuncture Research, Key Laboratory of Acupuncture and Neurology of Zhejiang Province, The Third Clinical Medical College, Zhejiang Chinese Medical University, Hangzhou, 310053 China

**Keywords:** Inflammatory pain, Electroacupuncture analgesia, Satellite glial cell, P2X7 purinergic receptor, P38 MAPK/TNF-α signaling

## Abstract

**Background:**

Accumulating evidence highlights the anti-inflammatory and analgesic effects of electroacupuncture (EA), yet the underlying mechanisms remain poorly understood. The P2X7 purinergic receptor (P2X7R), located in the peripheral and central nervous systems, has been implicated in the development of chronic inflammatory pain. Inhibition of P2X7R expression has been associated with the analgesic effects of EA. Within the dorsal root ganglia, P2X7R is exclusively expressed in satellite glial cells (SGCs), but its role in the anti-inflammatory effects of EA remains to be elucidated.

**Methods:**

A chronic inflammatory pain model was established in rats via intraplantar injection of Complete Freund’s Adjuvant (CFA). Western blotting, immunostaining, behavioral assay, pharmacological interventions, AAV-mediated knockdown assays, SGCs culture and real-time cell proliferation analysis were utilized to investigate the cellular mechanisms underlying the effects of EA at the ST36 and BL60 acupuncture points in mitigating inflammatory pain.

**Results:**

Rats injected with CFA exhibited long-lasting pain hypersensitivity in the ipsilateral hind paw, accompanied by upregulated expression of P2X7R and TNF-α in the L_4-6_ DRGs. Pharmacological inhibition or shRNA-mediated knockdown of P2X7R in SGCs significantly attenuated inflammatory pain hypersensitivity, reduced P38 MAPK phosphorylation, and decreased TNF-α levels. EA treatment significantly alleviated CFA-induced pain hypersensitivity and suppressed P2X7R expression alongside its downstream P38 MAPK/TNF-α signaling. The analgesic effects of EA were reversed by the P2X7R agonist BzATP. In vitro findings confirmed that TNF-α secretion by SGCs was markedly elevated in the CFA model but reduced by EA treatment, mimicking the effects of P2X7R antagonism.

**Conclusions:**

These findings demonstrate that the inhibition of P2X7R in SGCs and its downstream P38 MAPK/TNF-α signaling pathway contributes to the analgesic effects of EA in chronic inflammatory pain. Targeting peripheral P2X7R in SGCs may provide new insights into the cellular mechanisms of EA’s anti-inflammatory effects.

**Supplementary Information:**

The online version contains supplementary material available at 10.1186/s13020-025-01227-6.

## Introduction

Chronic pain conditions are intractable and remain difficult to manage. It presents a major humanistic burden and economic cost on society. Neuroinflammation, is a critical process that contributes to the initiation and maintenance of chronic inflammatory pain, which is considered one of the fundamental causes of chronic pain [1]. Pharmacological interventions, including corticosteroids and non-steroidal anti-inflammatory drugs (NSAIDs), form the cornerstone of anti-inflammatory therapy strategies. However, the use of these medications is limited due to their severe adverse effects on the cardiovascular, gastrointestinal, and renal systems [2–5], highlighting the need for novel therapeutic targets and approaches. The blood–brain barrier separates the central nervous system from the rest of the body, but dorsal root ganglia (DRG) tissues in the peripheral nervous system have unique vascularization and endothelial cells that allow drugs to permeate and accumulate in the tissue [6]. Restricting drug distribution to DRG tissues, bypassing the central nervous system, or targeting pain mechanisms that are exclusively expressed or enriched in primary sensory neurons, may help minimize side effects [7, 8]. To address chronic pain, novel analgesic approaches that target DRGs in the peripheral nervous system will become increasingly important in the future.

A growing body of research indicates that glial cells play a crucial role in the processes underlying chronic pain [9–11]. Satellite glial cells (SGCs), which surround DRG neurons, also play an active role in the pathophysiology and progression of chronic pain. Accumulating evidence suggests that under conditions of inflammation and nerve injury, SGCs become activated and undergo a series of functional changes, such as enhanced gap junction formation with neighboring SGCs or neurons, increased sensitivity to ATP, reduced function of the inwardly rectifying K channel subunit (Kir4.1), and increased cytokine release [12–16]. SGCs’ surface contains P2X7 purinergic receptor (P2X7R), P2Y12R, P2Y14R, N-methyl-D-aspartate receptor (NMDAR), glutamate-aspartate transporter (GLAST) and other receptors [12, 17–21]. Unlike other purinergic receptors, P2X7R is preferentially expressed in the DRG's SGCs and is involved in pain signaling, closely associated with the development and maintenance of inflammatory pain [21].

Several clinical studies have demonstrated that electroacupuncture (EA) produces significant anti-inflammatory and analgesic effects [22–24]. Recently, researchers have increasingly focused on understanding the mechanisms of EA. In mechanism studies, the treatment of inflammatory pain mainly focuses on peripheral modulation [25–27]. Recently, professor Qiufu Ma and his colleagues pointed out that EA stimulation of different intensities affected peripheral sympathetic NPY^+^ neurons, thus suppressing inflammatory responses [28, 29]. EA at the hindlimb ST36 acupuncture points counteracted the cytokine storm by evoking of the vagal-adrenal anti-inflammatory axis through PROKR2^Cre^-marked sensory neurons [28, 29]. Although EA has been shown to exert analgesic effects by inhibiting P2X7R expression in the DRG [30], direct evidence of EA’s effects on P2X7R regulation in activated SGCs remains unexplored. TNF-α, a known pain mediator in the peripheral nervous system [31], is implicated in inflammatory pain nociceptive sensitization [32]. In addition to this, rapid phosphorylation of P38 MAPK in the DRG is a hallmark of nociceptor activation [33], and its activation contributes to the development and maintenance of inflammatory pain [32]. It has been shown that CFA-induced inflammatory pain can be alleviated by down-regulating the phosphorylated expression of P38 MAPK [34]. While P2X7R, P38 MAPK, and TNF-α in DRGs are all involved in inflammatory pain's initiation, progression, or maintenance, their interrelationships remain unclear. It has been reported that SGCs can activate P38 MAPK through the production of cytokines such as TNF-α [35], and activated P38 MAPK can further enhance TNF-α release [36]. Inhibition of P2X7R reduces P38 MAPK phosphorylation expression and thus TNF-α expression to alleviate neuropathic pain [37], and antagonizing P2X7R function in a chronic postoperative pain model inhibits TNF-α elevation and thus exerts analgesic effects [38]. However, it remains unclear whether EA reduces P2X7R expression, thereby inhibiting the SGC-induced P38 MAPK/TNF-α signaling pathway to prevent inflammatory pain.

In this study, Complete Freund’s adjuvant (CFA) was injected into the plantar to establish a classic chronic inflammatory pain model in rats, and the potential analgesic mechanisms of EA involving P2X7R and P38 MAPK/TNF-α pathway in SGC were explored.

## Materials and methods

### Animals

Male Sprague–Dawley rats weighing 180–220 g were obtained from the Shanghai Experimental Animal Center of the Chinese Academy of Sciences [SCXK (Shanghai) 2018–0006]. The rats were housed at the Animal Research Center of Zhejiang Chinese Medical University [SYXK (Zhejiang) 2018–0012] under standard environmental conditions (room temperature: 24 ± 2 °C, humidity: 60 ± 5%, with a 12/12 h light–dark cycle) and had ad libitum access to water and food. All animal experiments were approved by the Ethics Committee of Zhejiang Chinese Medical University (IACUC Approval No. 20190715–03) and conducted in compliance with the relevant ethical guidelines for animal research. Sample sizes for animal experiments were determined in accordance with the 3R principles (Replacement, Reduction, and Refinement) and calculated using the statistical power analysis method described by Dell et al. [39]. This approach guarantees both scientific robustness and full ethical compliance across all experimental procedures.

### Inflammatory pain rat model establishment

After one week of adaptive feeding and baseline behavioral measurement, the inflammatory pain model was established using an intra-plantar injection of CFA, as previously described [40]. A 100 μL solution of CFA (F5881, Sigma, USA) was slowly injected into the plantar surface of the left hind paw, and the injection site was pressed for approximately 30 s to prevent leakage. For the control rats, an equivalent volume of normal saline (NS) was injected instead. Mechanical thresholds and thermal latencies were measured to confirm the success of the model.

### Electroacupuncture treatment

For EA treatment, rats in the CFA + EA group were restrained using a custom cotton restraint designed in our laboratory (Patent No. ZL 2014 2 0473579.9, State Intellectual Property Office of the People's Republic of China). Sterilized disposable stainless steel acupuncture needles (0.25 mm diameter, 13 mm length, Suzhou Medical Appliance Factory, Suzhou, China) were inserted bilaterally at a depth of 5 mm at ST36 (Zusanli, located 5 mm lateral to the anterior tubercle of the tibia) and BL60 (Kunlun, located at the level of the ankle joint, between the tip of the lateral malleolus and the Achilles tendon). The two homolateral needles were connected to two electrodes of the same output terminal at the FANGS Frequency Adjusting Nerve & General Stimulator (FANGS-100, Dalishen Medical Device Co., Ltd., Hangzhou, China).

The EA parameters were set as follows: dilation wave (pulse width: 0.4 ms at 2 Hz, 0.2 ms at 100 Hz, automatically alternating between 2 and 100 Hz stimulation every 3 s); 0.5–1.0–1.5 mA (each intensity applied for 10 min) for a total duration of 30 min. EA stimulation was administered daily for 30 min from day 8 to day 14 after CFA injection, for a total of seven sessions. For the sham EA treatment, rats received needle insertion subcutaneously at acupoints (1 mm depth) without electrical stimulation; all other procedures were identical to those in the EA group.

### Behavioral measurement

#### Von frey test

Mechanical allodynia was assessed using von Frey filaments (Stoelting Co., Thermo, Gilroy, CA, USA) following the up-down method described by Chaplan et al. [41]. Rats were placed in transparent cages for at least 15 min to acclimate prior to each test. Von Frey filaments of increasing stiffness (4, 6, 8, 15 and 26 g) were used to slowly and gently stimulate the center of the left hind paw for at least 5 s. The filament with the next incremental stiffness was applied based on the rat’s reaction to the previous stimulation. Following the first positive reaction, the sequence of responses was recorded after four stimulations. If the paw withdrew or flinched, a positive response was recorded with an "X". The force necessary for 50% withdrawal was calculated using the up-down method.

#### Hargreaves test

Rats underwent the Hargreaves test with a plantar tester (Ugo Basile 37,370, Italy) to determine paw withdrawal latencies (PWLs) [42]. Rats were acclimated to the experimental enclosures for 3 days, spending 60 min per day. On the testing day, each rat was acclimated in the testing enclosure for 60 min before being stimulated with a radiant heat beam applied to the center of the hind paw. The reaction time between the start of the thermal stimulation and the lifting of the hind paw was automatically recorded as the PWL. The test was repeated five times on the same hind paw with an interval of at least 5 min between each trial. The cut-off time was set at 20 s to prevent tissue damage. The average withdrawal latencies were used as the final PWLs.

#### Intrathecal injection

Intrathecal catheters were implanted 7 days before CFA injection, as previously described [43]. Under anesthesia with a mixture of oxygen and isoflurane (2–5% isoflurane in 100% oxygen) in rats, a sterile PE-10 polyethylene catheter filled with normal saline was inserted through the intervertebral space at L_5_/L_6_, with the other end passing through the neck and back. Additionally, the tube tip was positioned at the lumbosacral spinal level. Finally, penicillin (200,000 units) was administrated intramuscularly at the surgical site to prevent infection. Animals exhibiting postoperative hindlimb paralysis or paresis were excluded from the study. A 2% lidocaine solution was injected into the catheter to confirm the intraspinal location, and proper catheter placement was verified by immediate bilateral hindlimb paralysis (within 15 s), lasting 20–30 min. A740003 and BzATP were prepared as previously described [44]. Briefly, A740003 (Tocris Bioscience, United Kingdom), a selective P2X7R antagonist, was dissolved in DMSO and diluted to 250 nM/L in saline. BzATP (Sigma-Aldrich, USA), a selective P2X7R agonist, was dissolved in distilled water and diluted to 280 nM/L in saline. SB203580 (MedChemExpress, China), a selective p38MAPK inhibitor, was dissolved in DMSO and diluted to 1 μg /μl in saline [45]. All drugs were administered intrathecally on days 12–14 after CFA injection. Each 10 μL drug injection was followed by a 25 μL saline flush to ensure complete catheter delivery.

#### Intra-DRG injection

The shRNA was injected into the ipsilateral L_4_ DRG of rats approximately 3 to 4 weeks before CFA or NS injection. The intra-DRG injection protocol was modified based on the method described by Gregory Fischer et al. [46]. The rats were anesthetized with pentobarbital sodium (40 mg/kg, i.p.). The rats were placed in a prone position, with the spine fixed in a stereotactic frame. A 2 cm longitudinal incision was made to expose the L_4_ and L_5_ vertebrae, after which the multifidus and longissimus lumborum muscles were removed. The articular process and portions of the lumbar transverse process on the left side were removed to expose the L_4_ DRG. ShRNA (3 μL per DRG) was injected at a speed of 20 nL/s using a glass microelectrode in the micro-syringe operated by a micropump (World Precision Instruments, Sarasota, USA). rAAV2/5-GFaABC1D-cre-mcherry- WPRE-hGH and rAAV2/9-CMV-DIO-(mCherry-U6)-shRNA (scramble)-WPRE-Hgh (scr-shRNA for short) were injected into the ipsilateral L_4_ DRG of the Con or CFA group. rAAV2/5-GFaABC1D-cre-mcherry-WPRE-hGH and rAAV2/9-CMV-DIO-(mCherry-U6)-shRNA(P2X7R)-WPRE-hGH (P2X7R-shRNA for short) were injected into the ipsilateral L_4_ DRG of the CFA group. Penicillin (200,000 units, i.m.) was administrated immediately after surgery.

#### Dorsal root ganglion neuron dissociation and cell culture

Rats were sacrificed on day 14, and the bilateral or ipsilateral L_4-6_ DRGs were removed from the control (Con) group and other groups respectively, and then transplanted into Hank's buffered saline solution (HBSS, H2387, Sigma Aldrich, St. Louis, MO, United States) containing 10 mM HEPES. The L_4-6_ DRGs were collected and dissociated with 1 mg/mL collagenase type 1 and 2 mg/mL dispase (Gibco, Thermo Fisher Scientific, USA) for 40 min, followed by 12 min at 37 °C in 0.25% trypsin (T4549, Sigma Aldrich, St.Louis, MO, United States). After washing with DMEM/F12 plus 10% FBS (FBS, 1,828,728, Gibco, United States), 100 units/mL penicillin and 100 μg/mL streptomycin, cells were dissociated using a fire-polished glass Pasteur pipette. The dissociated cells were then plated on Matrigel-coated dishes (354,234, Corning, NY, United States), and the cultures were kept humid at 37 °C with 5% CO_2_.

#### Satellite glial cell enriched cultures

DRG cell suspension was cultured in vitro for four days when SGCs were abundant. 1 mL of 0.25% trypsin was added to isolate SGCs for 3 min before discarding. After adding 1 mL of fresh culture medium (DMEM/F12 containing 10% FBS, 100 units/mL penicillin and 100 μg/mL streptomycin), cells were gently removed from the culture dishes using a fire-polished Pasteur pipette. After collecting1 mL of cell suspension, it was centrifuged at 1000 rpm for 5 min at room temperature to precipitate the cells. 250 µL of the cell suspension was then plated on new uncoated 24-well plates. Untreated dishes prevent the attachment of neurons but have no effect on the attachment or growth of the glial cells [47]. Furthermore, the culture medium was changed 2 h after the cells were plated to eliminate any cells (mostly neurons) in suspension. SGC-enriched cultures were incubated in a humid 5% CO_2_ atmosphere at 37 ℃ for 3 days. The culture medium was replaced every other day. SGCs used in immunocytochemistry assays were plated on uncoated slides and processed in vitro after 3 days to allow for SGC growth.

#### Real-time cell proliferation analysis(RTCA)

Cell proliferation was assessed by real-time cell counting using an xCELLigence system (DP system, ACEA Biosciences, San Diego, CA, USA) and E plates (Roche), which monitors cellular events in real time by measuring the electrical impedance across interdigitated gold microelectrodes integrated into the bottom of tissue culture plates. Impedance measurement provides quantitative information about the cell number [48–50]. SGCs from the Con, CFA and CFA + EA groups were cultured and enriched. Each well of SGCs in the CFA group received 100 μL of a medium solution containing A740003 at a final concentration of 1 μM. Cellular events were monitored for 24 hours. Thirty minutes before the end of the experiment, 100 μL of a medium solution containing BzATP at a final concentration of 100 μM was added to each well of SGCs in the CFA + EA group. Cellular events were then recorded for 30 min.

#### Enzyme-linked immunosorbent assay (ELISA)

L_4-6_ DRGs were collected and homogenized at full speed for 20 min using a Bullet Blender (NextAdvance, USA) in 50 mM Tris-base (pH 7.4) and 150 mM NaCl with protease inhibitor (#04693132001, Roche, Switzerland) and 0.2% Triton-X. The homogenates were centrifuged at 10,000 rpm for 15 min at 4 °C. The cell suspension was centrifuged at 10,000 rpm for 15 min, and the supernatant was immediately collected for detection. The supernatant was tested for TNF-α level using an ELISA kit (R&D Systems, USA) according to the manufacturer's instructions. The plates were analyzed at 450, 540, and 570 nm using a microplate reader. Total protein was determined by the BCA assay (Thermo Fisher Scientific, USA).

#### Immunofluorescence

For immunofluorescence of DRG, The DRGs were fixed with 4% paraformaldehyde and dehydrated with sucrose gradients for 3 days. After dehydration, the samples were sectioned at 12 μm using a cryostat (CryoStar, NX50, Thermo Fisher, USA). The sliced sections were then mounted onto gelatin-coated glass slides. The sections were blocked with 5% donkey serum in TBST (with 0.3% Triton X-100, blocking buffer) for 1 h at 37 °C. For immunocytochemistry, after washing with 4% paraformaldehyde solution, the cells were permeabilized for 30 min at room temperature using a 0.3% Triton X-100 solution diluted in PBS solution, before being blocked with 3% BSA diluted in PBS solution for 1 h at room temperature. And then incubated with the following primary antibodies at 4 ℃ overnight: P2X7R antibody (1:400, rabbit polyclonal, GTX), GFAP antibody (1:400 for DRG, 1:200 for cells, mouse monoclonal, Abcam), NeuN antibody (1:400, mouse monoclonal, Abcam), Iba1 antibody (1:400, mouse monoclonal, Abcam). Secondary antibodies, including Donkey Anti-Rabbit IgG H&L (Alexa Fluor® 488), Donkey Anti-Mouse IgG H&L (Alexa Fluor® 594), and Donkey Anti-Mouse IgG H&L (Alexa Fluor® 488) (Abcam), were incubated with the sections for 1 h at 37 °C, followed by the addition of an appropriate amount of anti-fluorescence quencher containing DAPI. The stained and mounted sections were then inspected using a Zeiss Apotome 3 microscope (Zeiss, Germany). Images were captured using ZEN software (Zeiss, Germany).

#### Western blotting

Rats were deeply anesthetized with pentobarbital sodium (40 mg/ kg; i.p.). Ipsilateral L_4_-_6_ DRGs were harvested, weighed, and homogenized in RIPA buffer with protease and phosphatase inhibitors. The supernatant was collected after the lysate was centrifuged for 10 min at 4 °C at 14,000 rpm. The protein content was then ascertained using the bicinchoninic acid (BCA) method with a constant protein loading volume (20 μg), following the manual instructions (Thermo Fisher Scientific, USA). Samples were separated by SDS-PAGE, transferred to PVDF membranes, and then incubated with primary antibodies overnight at 4 °C after being blocked for 1 h at room temperature with 5% non-fat milk in TBS containing 0.1% Tween-20 (pH 7.5). After washing, membranes were incubated with HRP-conjugated secondary antibodies (1:5000) for 1 h at 37 °C. Staining was visualized using enhanced chemiluminescence detection reagents, followed by exposure to FluorChem Protein Simple (AlphaImager ProteinSimple, San Jose, CA, USA), and analyzed using ImageJ software, with GAPDH as the loading control. The primary antibodies used are as follows: rabbit anti-P2X7R (1:1000, Alomone), rabbit anti-P38 MAPK (1:1000, CST), rabbit anti-pP38 MAPK (1:1000, CST), GAPDH (1:1000, CST).

#### Statistical analysis

All data were presented as mean ± SD and analyzed using GraphPad Prism8.0 (GraphPad Software Inc., San Diego, CA, USA). Data normality was verified using the Shapiro–Wilk test. The comparison between two groups was performed by Student’s *t* test (two-tailed). One-way or two-way ANOVA followed by Tukey’s post hoc test was used for comparison among three or more groups. ANOVA with repeated measures was performed when necessary. Significant differences between comparisons were defined as *p* < 0.05.

## Results

### Intraplantar injection of CFA induced pain hypersensitivity and upregulated P2X7R, P38 MAPK phosphorylation and TNF-α in DRG.

**Fig. 1:**
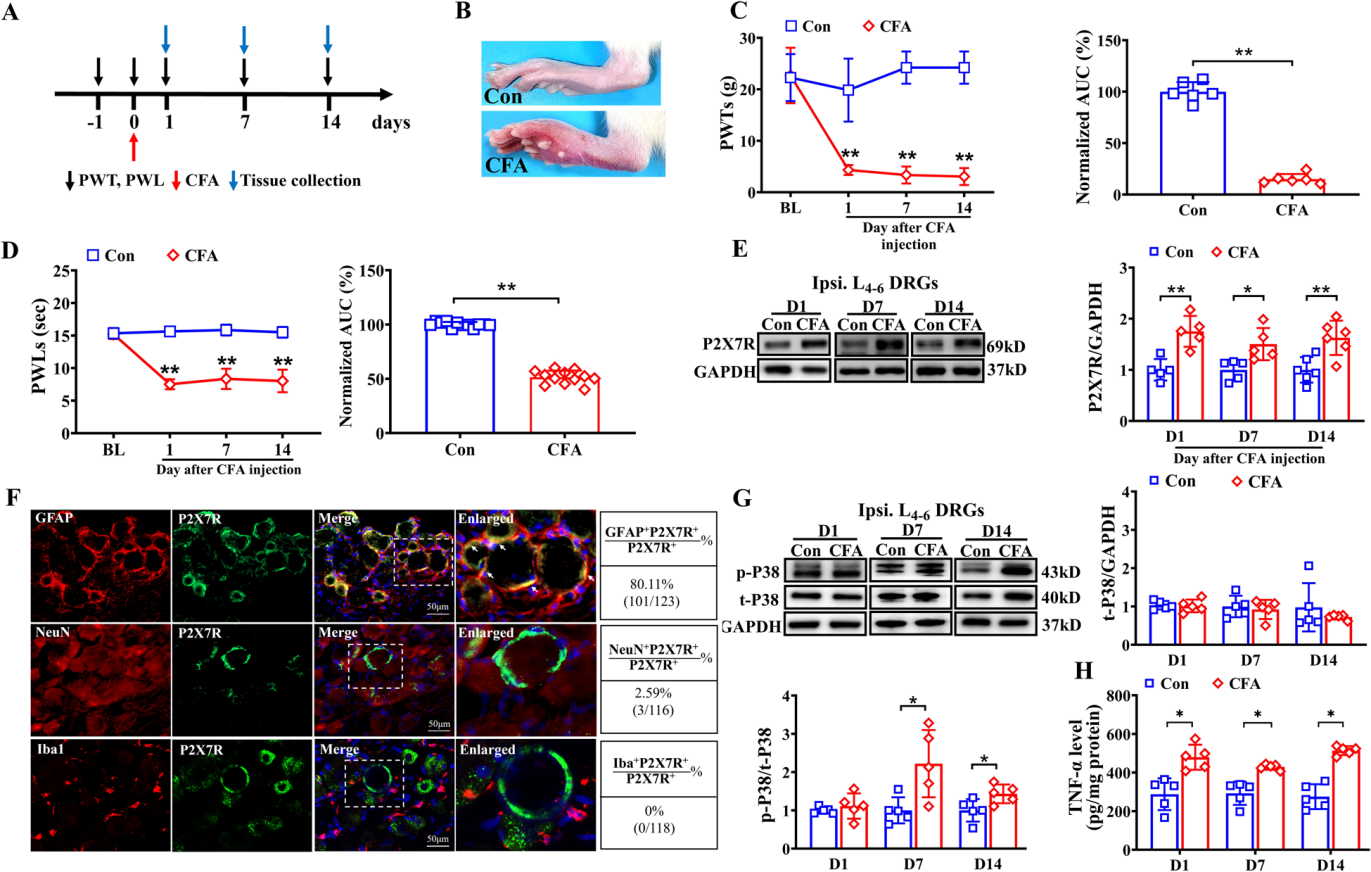
CFA rat showed persistent pain hypersensitivity, accompanied with P2X7R, P38 MAPK phosphorylation and TNF-α upregulation in ipsilateral DRGs. **A** Diagram showing the experiments’ design. **B** Representative graph of normal paw (upper) and CFA-induced inflamed paw (lower) at D7 post-injection. **C** PWTs measured in ipsilateral hindpaw before and after CFA injection (left) and normalized area under the curve (AUC) analysis of PWTs (right), n = 6/group. **D** PWLs measured in ipsilateral hindpaw before and after CFA injection (left) and AUC analysis of PWLs (right), n = 12/group. **E** Western blot showing P2X7R expression in ipsilateral L_4-6_ DRGs of Con and CFA group on D1, 7 and 14, n = 5–6 rats/group. **F** Double immunostainings of P2X7R with GFAP (satellite glial cells marker, top row), NeuN (neuron marker, middle row) or Iba1 (macrophages marker, bottom row) in L_4_ DRG sections of CFA rats on D14. Quantification of cellular distribution was shown on the right (pooled from 4 rats/group). Scale bar indicates 50 μm. **G** Western blot showing the expression of P38 MAPK and its phosphorylation (Thr180/Tyr182) in the Con and CFA rats on D1, 7, 14 respectively. n = 5 rats/group. **H** TNF-α levels in ipsilateral DRG of Con and CFA rats on D1, 7, 14 detected by ELISA. n = 5 rats/group. *p < 0.05, **p < 0.01 vs. Con group. Two-way ANOVA with repeated measures followed by Tukey’s post hoc test was used for comparisons in (**C**) (left), (**D**) (left). Student’s t test was used for comparisons in (**C**) (right), (**D**) (right), (**E**, **G** and **H)**

A rat model of inflammatory pain was successfully established based on a previously reported protocol, with nociceptive behaviors systematically measured. On day 7 post-injection, the CFA group showed obvious inflammatory signs (redness, swelling, heat, and pain) in the ipsilateral paw. Visible paw swelling, increased circumference, and tense, glossy skin were observed, with heightened sensitivity to touch. No such changes occurred in the control group (Fig. 1B). Significant mechanical allodynia, evidenced by a notable decrease in 50% paw withdrawal thresholds (PWTs), was observed from Day 1 (D1, 24 h after CFA injection) following the injection and persisted until the end of the observation period on Day 14 (D14) in CFA-treated rats compared to control (Con) rats (Fig. 1C). Similarly, thermal hyperalgesia, characterized by a reduction in paw withdrawal latencies (PWLs), was evident from D1 (24 h after CFA injection) and persisted through D14 (Fig. 1D). Area under the curve (AUC) analysis further confirmed the accumulation of mechanical allodynia and thermal hyperalgesia (Fig. 1C and D), validating the successful induction of inflammatory pain in the rats.

Previous studies have demonstrated that P2X7R expression in the DRG is activated in response to peripheral inflammation [21]. In this study, Western blot analysis was used to evaluate changes in P2X7R expression in ipsilateral L_4-6_ DRGs on D1 (24 h after CFA injection), 7 and 14 following CFA injection. The results showed a significant increase in P2X7R protein expression in ipsilateral L_4-6_ DRGs in CFA-treated rats compared to Con rats across all time points (Fig. 1E). These findings indicate that CFA-induced inflammatory pain markedly increases P2X7R expression in the L_4-6_ DRGs, with elevated levels persisting for at least 14 days post-injection. Immunofluorescence staining demonstrated P2X7R was co-expressed with SGCs (marked by GFAP), but not with neurons (marked by NeuN) or macrophages (marked by Iba1) in DRGs (Fig. 1F). We further found that phosphorylated P38 MAPK (p-P38 MAPK) and TNF-α expressions were significantly increased in DRGs during chronic inflammatory pain, and sustaining through day 14 post-CFA injection, whereas total P38 MAPK levels remained unchanged (Fig. 1G, H).

### Antagonism of P2X7R reduced inflammatory pain, activation of SGCs, P38 MAPK phosphorylation and TNF-α levels in the DRG of CFA rats

**Fig. 2:**
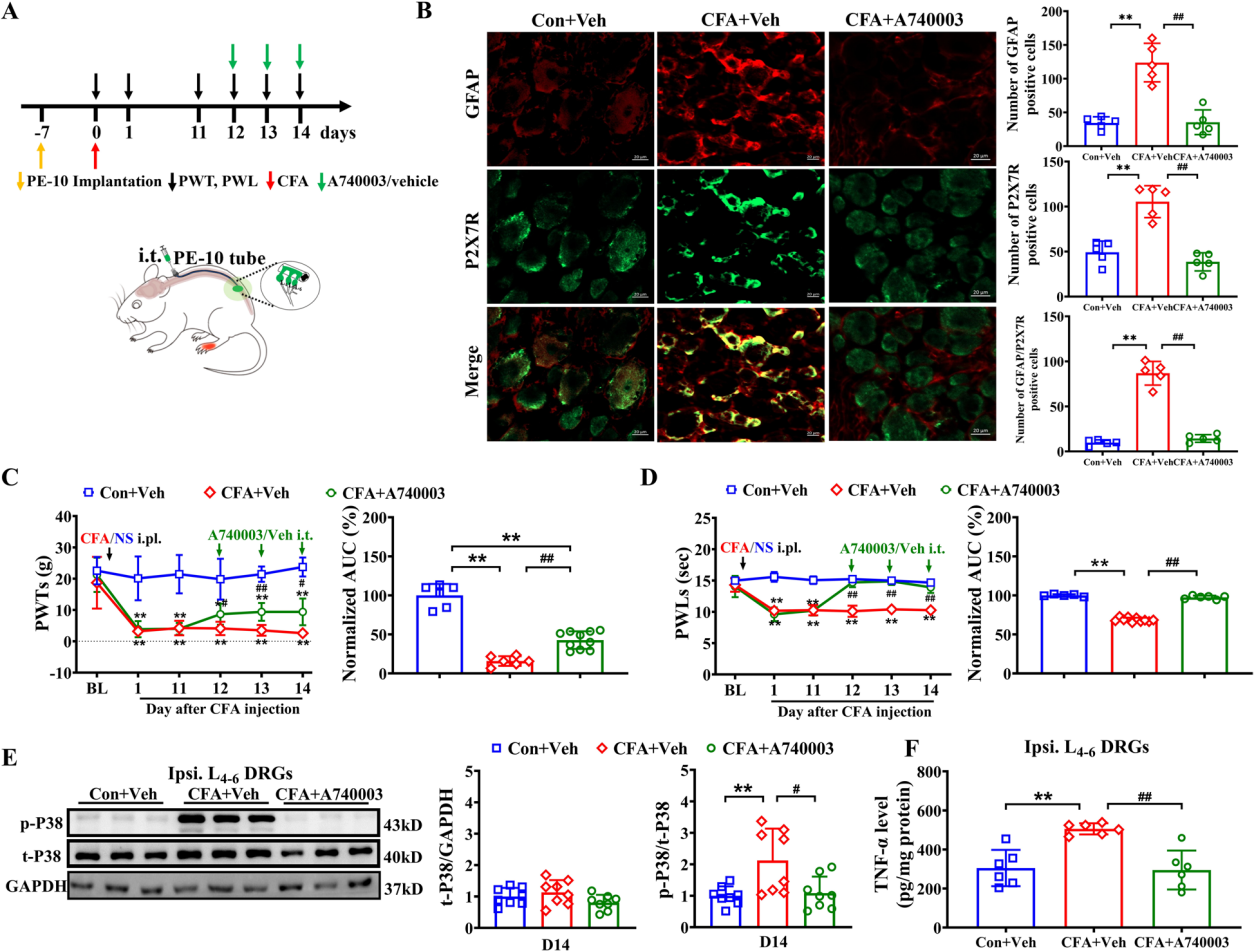
Pharmacological blocking P2X7R inhibited pain hypersensitivity, activation of SGCs, phosphorylation of P38 MAPK and TNF-α levels. **A** Experimental diagram illustrating specific time points for CFA model establishment, intrathecal injections of P2X7R antagonist or vehicle, and behavioral test. **B** Representative immunofluorescence pictures showing the co-localization of P2X7R with GFAP. The bar graphs indicate the number of CFAP positive cells, P2X7R positive cells, and GFAP/P2X7R positive cells in each group's DRG. Scale bar indicates 20 μm, n = 5/group. **C** Time course showing the effect of P2X7R antagonist A740003 (250 nmol/L in 10 μL) on mechanical allodynia in CFA rats (left) and summary of normalized AUC of PWTs curve (right), n = 5–10/group. **D** Time course showing the effect of A740003 on thermal hyperalgesia in CFA rats (left) and summary of normalized AUC analysis of PWLs (right), n = 5–10/group. **E** Western blot showing the expression of P38 MAPK and its phosphorylation (Thr180/Tyr182) in the Con + Veh, CFA + Veh and CFA + A740003 groups on D14. n = 8 rats/group. **F** TNF-α levels in ipsilateral L_4-6_ DRGs of Con + Veh, CFA + Veh and CFA + A740003 rats on D14. n = 6 rats/group. **p < 0.01 vs Con + Veh group; #*p* < 0.05, ##*p* < 0.01 vs. CFA + Veh group. Two-way ANOVA with repeated measures followed by Tukey’s post hoc test was used for comparisons in C (left), D (left). One-way ANOVA followed by Tukey’s post hoc test was used for comparisons in others

To investigate P2X7R's role in CFA-induced pain hypersensitivity, we administered the selective antagonist A740003 or vehicle via intrathecal injection at predetermined time points (Fig. 2A). While A740003 showed no effect on baseline pain thresholds in naïve rats (Supplemental Fig. 1A, B), it significantly attenuated ipsilateral mechanical allodynia and thermal hyperalgesia in CFA rats compared to vehicle controls (Fig. 2C, D). The immunofluorescence results demonstrated that compared with the Con + Veh group, the CFA + Veh group exhibited significantly increased numbers of P2X7R-positive cells and enhanced SGC activity in the ipsilateral DRG. Intrathecal injection of A740003 markedly suppressed the CFA-induced overexpression of both P2X7R and SGC in the ipsilateral DRG (Fig. 2B). Additionally, we observed that A740003 injection (i.t.) markedly reduced the increase in phosphorylated P38 MAPK in the DRGs (Fig. 2E). ELISA results demonstrated that TNF-α level was significantly higher in the L_4-6_ DRGs of CFA + Veh rats compared to the Con + Veh group. Intrathecal injection of A740003 significantly reduced TNF-α levels in CFA rats (Fig. 2F). These findings indicate that P2X7R activation triggers SGC activation, increases P38 MAPK phosphorylation and TNF-α production, all of which contribute to chronic inflammatory pain.

### Knockdown SGCs’ P2X7R attenuated CFA-induced inflammatory pain via by inhibiting P38 MAPK/TNF-α pathway

**Fig. 3:**
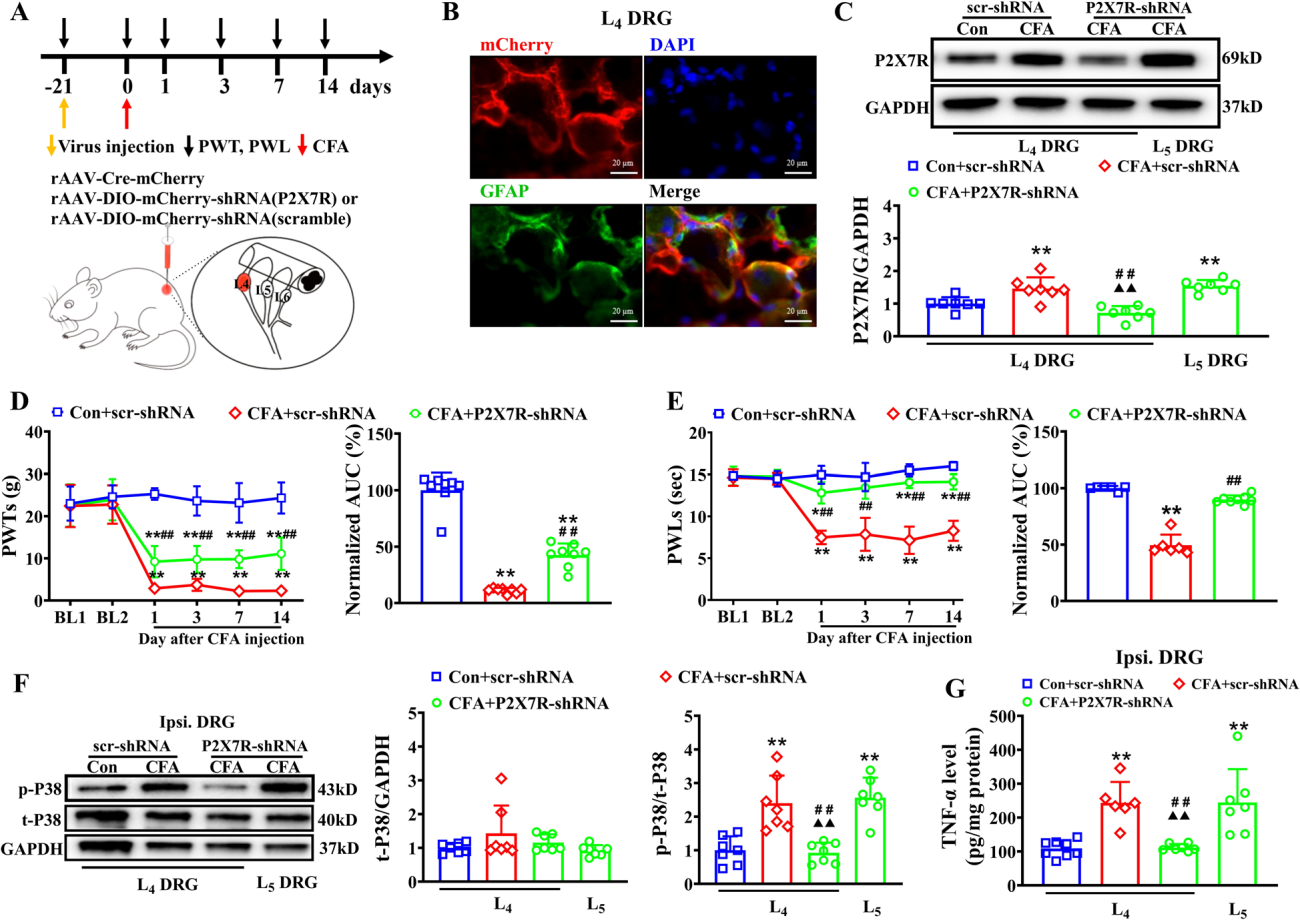
Knockdown P2X7R in the SGCs of L_4_ DRG alleviated CFA-induced inflammatory pain. **A** Experimental protocol showing the time points for CFA model establishment, intra-ganglia injections of virus and behavioral test (upper). Cartoon showing the injection of rAAV-Cre-mCherry and rAAV-DIO-mCherry-shRNA (P2X7R-shRNA) or scramble shRNA (scr-shRNA) into the L_4_ DRG of rats (lower). **B** Representative immunohistochemical images of L_4_ DRG at week 5 after intra-ganglia injection of virus. **C** Western blot showing P2X7R expression in ipsilateral L_4_ and L_5_ DRG at week 5 after virus injection. n = 7/group. **D** Time course showing the effect of P2X7R-shRNA on ipsilateral PWTs after CFA plantar injection (left) and summary of normalized AUC of PWTs curve (right). n = 8/group. **E** Time course showing the effect of P2X7R-shRNA on ipsilateral PWLs after CFA plantar injection (left) and summary of normalized AUC of PWLs curve (right). n = 6–8/group. **F** Western blot showing the expression of P38 MAPK and its phosphorylation (Thr180/Tyr182) in ipsilateral L_4_ and L_5_ DRGs of CFA + shRNA-P2X7R group and in ipsilateral L_4_ DRG of other groups. Representative blot images (left) and pooled data (right) had shown. n = 7/group. **G** TNF-α levels in ipsilateral DRG of rats among three groups detected by ELISA. n = 6–8/group. **p < 0.01 vs. Con + scr-shRNA group; ##*p* < 0.01 vs. CFA + scr-shRNA group; (double Filled triangle) *p* < 0.01 vs. CFA + P2X7R-shRNA(L_5_). Two-way ANOVA with repeated measures followed by Tukey’s post hoc test was used for comparisons in (**D**) (left), (**E**) (left). One-way ANOVA followed by Tukey’s post hoc test was used for comparisons in others

Since intrathecal A740003 administration might non-selectively modulate P2X7R-expressing cells in both DRGs and central nervous system, we employed a targeted genetic approach to specifically investigate the role of SGCs' P2X7R in the pathogenesis of inflammatory pain. We microinjected recombination adeno-associated virus (rAAV) expressing the *P2* × *7r* gene driven by the SGC promoter GFaABC1D into DRGs to achieve selective P2X7R knockdown in SGCs (Fig. 3A). The intra-DRG injection ensured localization of viral infection within the DRG region without affecting the spinal dorsal horn or other higher brain regions. As shown in Fig. 3B, 3 weeks after intra-DRG injection, rAAV2/5- GFaABC1D-mcherry was clearly expressed in GFAP-labeled SGCs, confirming the specificity of viral infection. Western blot analysis demonstrated a significant reduction in P2X7R protein levels in the L_4_ DRG of the CFA + P2X7R-shRNA group compared to both the CFA + scr-shRNA group and the L_5_ DRG of the CFA + P2X7R-shRNA group (Fig. 3C).

Behavioural analyses revealed that both PWTs and PWLs were significantly increased in CFA + P2X7R-shRNA rats compared to CFA + scr-shRNA rats at all observed time points following CFA injection. AUC analysis further indicated the cumulative attenuation of mechanical allodynia and thermal hyperalgesia in CFA + P2X7R-shRNA rats compared to CFA + scr-shRNA rats (Fig. 3D, E).

Western blot analysis showed a significant decrease in p-P38 MAPK levels, but not in total P38 MAPK levels, in the ipsilateral L_4_ DRG of CFA + P2X7R-shRNA rats compared to CFA + scr-shRNA rats. However, both phosphorylated and total P38 MAPK level remained unchanged in the L_5_ DRG of CFA + P2X7R-shRNA rats compared to CFA + scr-shRNA rats (Fig. 3F). ELISA results further demonstrated that TNF-α levels in the L_4_ DRG of the CFA + P2X7R-shRNA group were significantly lower than those in the L_5_ DRG or the CFA + scr-shRNA group (Fig. 3G). These results suggest that knockdown of SGCs’ P2X7R attenuates CFA-induced inflammatory pain via the P38 MAPK/TNF-α pathway.

### Inhibition of p38 Activation reduced inflammatory pain and TNF-α levels in the DRG of CFA rats

**Fig. 4:**
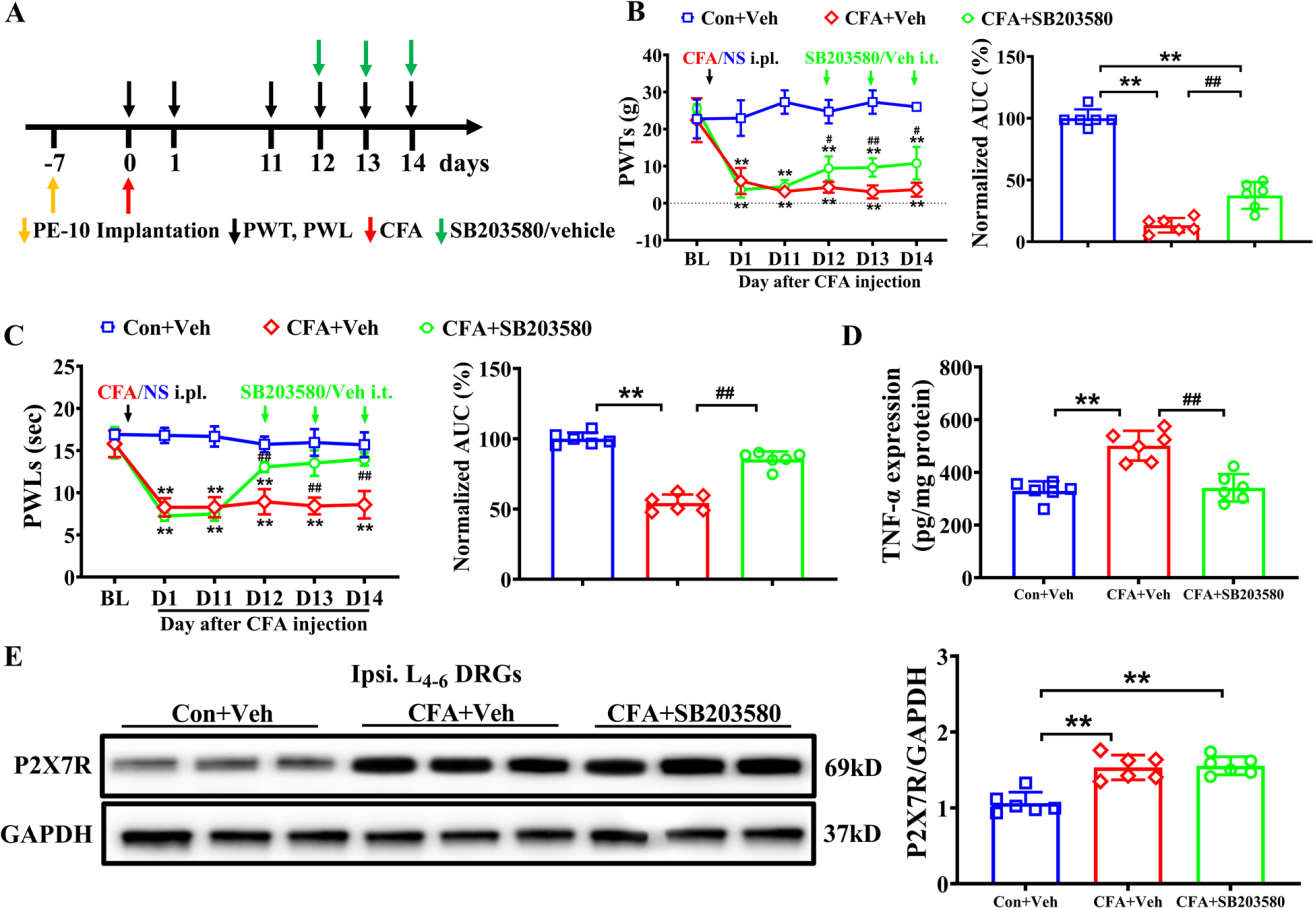
Pharmacological blocking P38 MAPK inhibited pain hypersensitivity and TNF-α levels. **A** Experimental diagram illustrating specific time points for CFA model establishment, intrathecal injections of p38 inhibition or vehicle, and behavioral test. **B** Time course showing the effect of p38 inhibition SB203580 (1 μg/μL in 10 μL) on mechanical allodynia in CFA rats (left) and summary of normalized AUC of PWTs curve (right), n = 6/group. **C** Time course showing the effect of SB203580 on thermal hyperalgesia in CFA rats (left) and summary of normalized AUC analysis of PWLs (right), n = 6/group. **D** TNF-α levels in ipsilateral L_4-6_ DRGs of Con + Veh, CFA + Veh and CFA + SB203580 rats on D14. n = 6/group. **E** Western blot showing the expression of P2X7R in the Con + Veh, CFA + Veh and CFA + SB203580 groups on D14. n = 6/group. **p < 0.01 vs Con + Veh group; #*p* < 0.05, ##*p* < 0.01 vs. CFA + Veh group. Two-way ANOVA with repeated measures followed by Tukey’s post hoc test was used for comparisons in (**B**) (left), (**C**) (left). One-way ANOVA followed by Tukey’s post hoc test was used for comparisons in others

To determine whether p38 MAPK participates in mechanical allodynia and thermal hyperalgesia in CFA rats and its relationship with P2X7R, we employed the same method as described earlier, intrathecally administering the p38 inhibitor SB203580 or its vehicle (Fig. 4A). Compared with the vehicle group, intrathecal injection of SB203580 significantly alleviated ipsilateral mechanical allodynia and thermal hyperalgesia in CFA rats (Fig. 4B, C) and markedly reduced TNF-α levels (Fig. 4D). However, P2X7R overexpression was not reversed (Fig. 4E). These results suggest that CFA-induced P2X7R expression in the DRG is independent of p38 phosphorylation, whereas CFA-triggered TNF-α upregulation in the DRG can be suppressed by inhibiting p38 activation, implicating p38 MAPK as a downstream mediator of P2X7R in mediating CFA-induced inflammatory pain.

### EA attenuated CFA-induced inflammatory pain via inhibiting P2X7R and P38 MAPK/TNF-α pathway

**Fig. 5:**
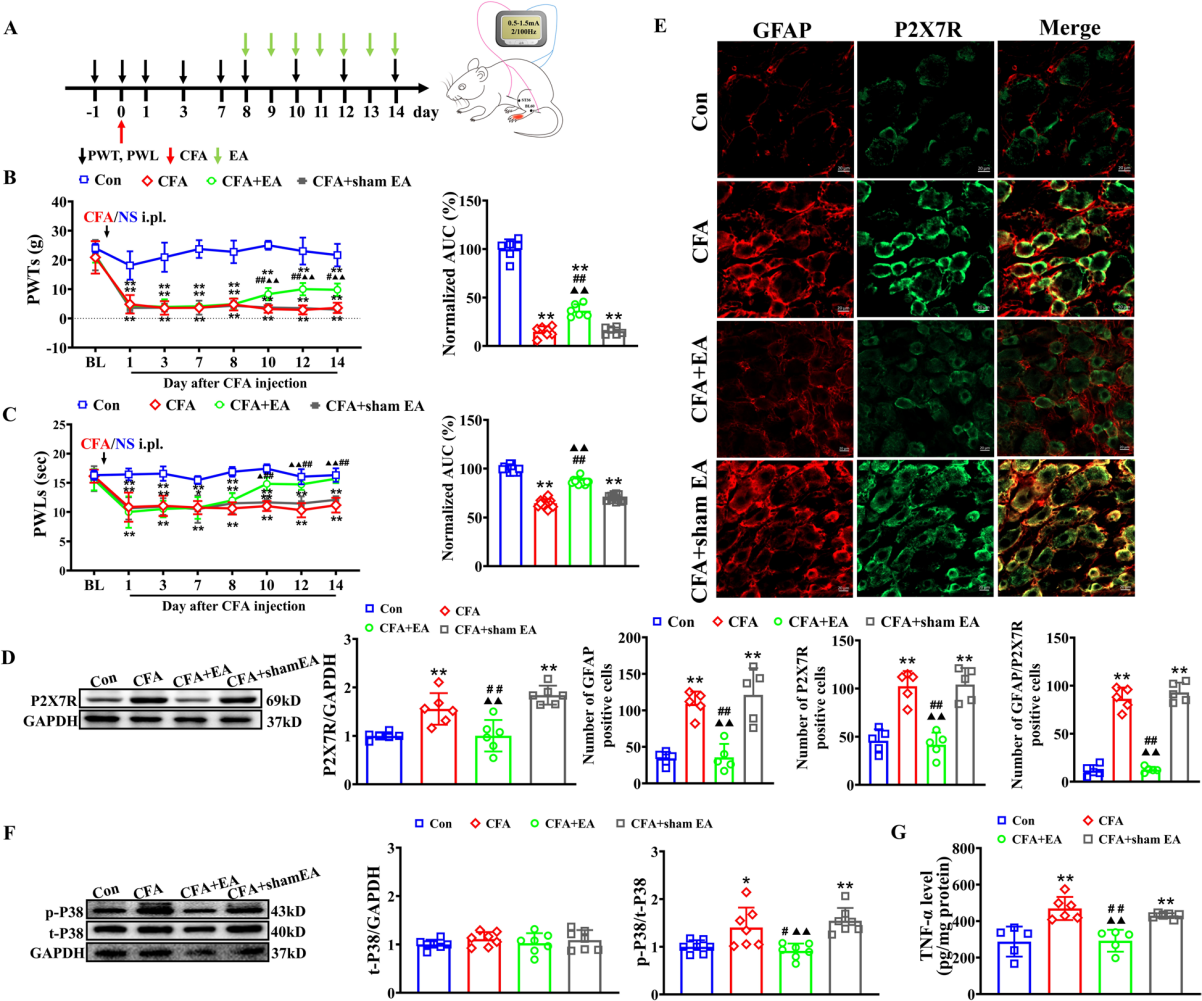
EA alleviated CFA-induced inflammatory pain as well as the activation of P2X7R-P38 MAPK/TNF-α signaling. **A** Experimental protocol illustrating time points for model establishment, EA/sham EA treatment and behavioral test (left). Cartoon illustrating the location of ST36 (5 mm lateral to the anterior tubercule of the tibia) and BL60 (at the ankle joint level and between the tip of the external malleolus and calcaneus) acupoints in the rat. EA treatment given daily from D8 to D14 at bilateral ST36 and BL60 acupoints with 2/100 Hz frequency, 0.5–1.0–1.5 mA intensity (10 min in each, 30 min in total) (right). **B** Time course showing the effect of 2/100 Hz EA treatment on mechanical allodynia in CFA rats (left) and summary of normalized AUC of PWTs curve from D8 to D14 (right). n = 6/group. **C** Time course showing the effect of 2/100 Hz EA treatment on thermal hyperalgesia in CFA rats (left) and summary of normalized AUC of PWLs from D8 to D14 (right). n = 12/group. **D** Western blot showing P2X7R expression in ipsilateral L_4-6_ DRGs of all four groups. n = 6/group. **E** Representative immunofluorescence pictures showing the co-localization of P2X7R with GFAP. The bar graphs indicate the number of CFAP positive cells, P2X7R positive cells, and GFAP/P2X7R positive cells in each group's DRG. Scale bar indicates 20 μm, n = 5/group. **F** Western blot showing the expression of P38 MAPK and its phosphorylation (Thr180/Tyr182) in ipsilateral L_4-6_ DRGs of all four groups. n = 7/group. **G** ELISA assay showing TNF-α level in ipsilateral L_4-6_ DRGs of all four groups. n = 5–6 rats/group. *p < 0.05, **p < 0.01, vs. Con group; #*p* < 0.05, ##*p* < 0.01 vs. CFA group; (Filled triangle) *p* < 0.05, (double Filled triangle) *p* < 0.01, vs. CFA + sham EA group. Two-way ANOVA with repeated measures followed by Tukey’s post hoc test was used for comparisons in B (left), C (left). One-way ANOVA followed by Tukey’s post hoc test was used for comparisons in others

To observe the effect of EA on CFA-induced inflammatory pain, we applied 2/100 Hz EA at bilateral ST36 and BL60 acupoints located on the hind limbs of the rats, starting from Day 8 to Day 14 after CFA injection (Fig. 5A). The 7-session EA protocol showed no significant effect on motor function as assessed by open field testing (Supplemental Fig. 2). At baseline, no significant difference in PWTs and PWLs were observed among groups. Compared to the Con rats, CFA rats exhibited a significant decrease in ipsilateral PWTs and PWLs from Day 1 to Day 14 post-CFA injection. Ipsilateral PWTs and PWLs in the CFA + EA group were higher than those in the CFA and CFA + sham EA groups during EA intervention. AUC analysis further confirmed that EA exerted a cumulative attenuation of mechanical allodynia and thermal hyperalgesia. As a non-pharmacological intervention, EA exhibits time-limited analgesic effects in CFA rats (Supplemental Fig. 3), consistent with clinical observations of its transient therapeutic duration.

**Fig. 6:**
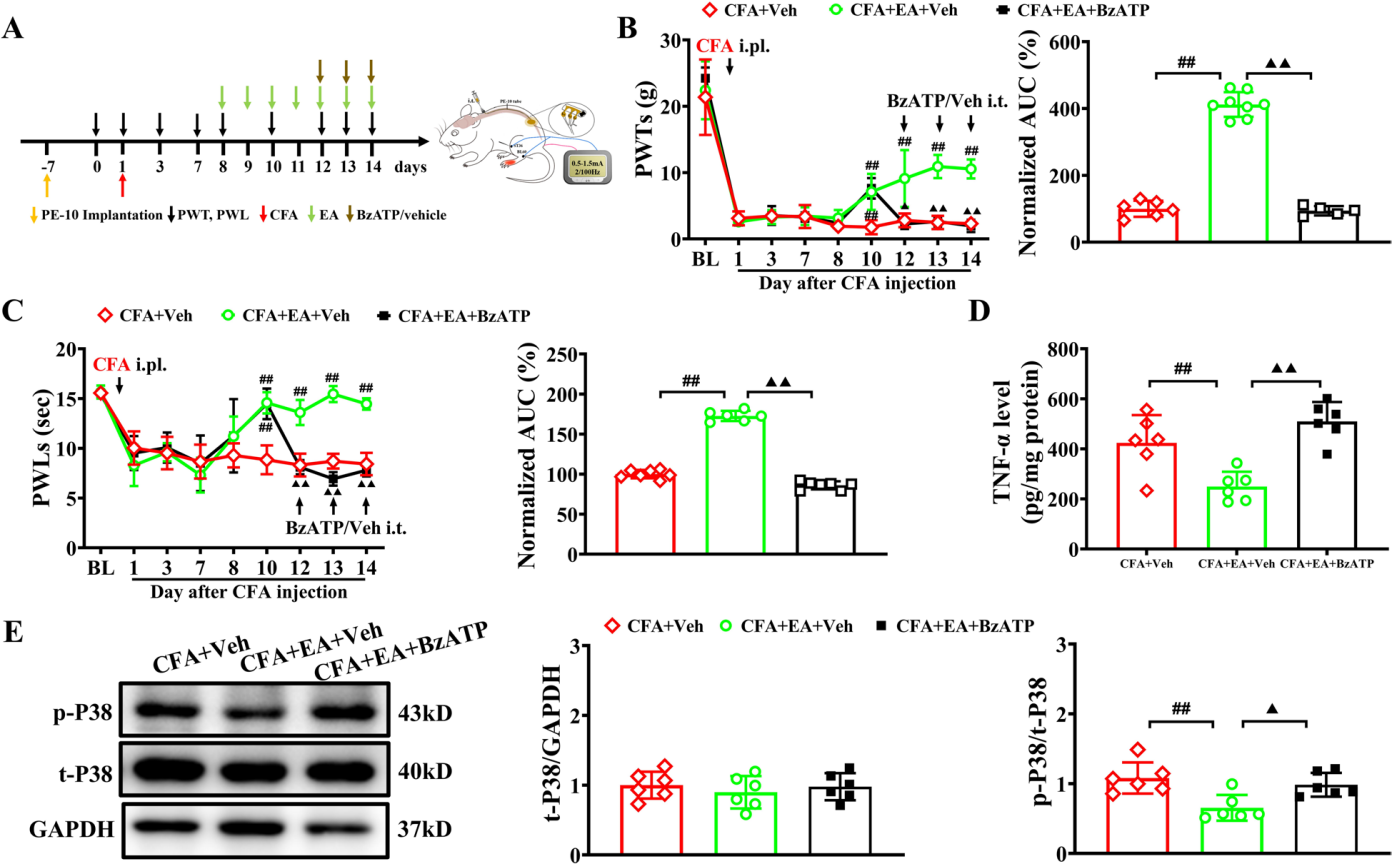
P2X7R agonist BzATP reversed the EA's analgesic effects on CFA-induced pain hypersensitivities. **A** Experimental protocol illustrating time points for behavioral test, EA stimulation and intrathecal injection of BzATP (280nmoL/L, 10μL) or vehicle. **B-C** Time course effect of BzATP on EA's analgesic effect on mechanical allodynia (**B**) or thermal hyperalgesia (**C**) in CFA rats (left). Summary of normalized AUC of PWTs or PWLs curve from D12 to D14 after CFA-injection (right). n = 6–8 rats/group. **D** ELISA assay showing TNF-α level in ipsilateral L_4-6_ DRGs of all three groups. n = 6 rats/group. **E** Western blot showing the expression of P38 MAPK and its phosphorylation (Thr180/Tyr182) in ipsilateral L_4-6_ DRGs of all three groups. n = 6 rats/group. ## *p* < 0.01, vs. CFA + Veh group; (Filled triangle) *p* < 0.05, (double Filled triangle) *p* < 0.01, vs. CFA + EA + Veh group. Two-way ANOVA with repeated measures followed by Tukey’s post hoc test was used for comparisons in (**B**) (left), (**C**) (left). One-way ANOVA followed by Tukey’s post hoc test was used for comparisons in others

To investigate whether EA’s analgesic effects were associated with the inhibition of the P2X7-mediated P38 MAPK/TNF-α pathway, we studied the effects of EA on P2X7R overexpression and the activation of the P38MAPK/TNF-α pathway. Additionally, we examined the influence of activating P2X7R using a selective agonist on EA's analgesic efficacy. Repeated EA stimulation at bilateral ST36 and BL60 acupoints significantly reduced P2X7R overexpression in the L_4–6_ DRGs of CFA rats, whereas sham EA showed no such effect (Fig. 5D). Immunofluorescence further analysis revealed that compared with the CFA group and the CFA + sham EA group, EA treatment significantly reduced the number of P2X7R-positive cells in the ipsilateral DRG, accompanied by decreased SGC activation and reduced P2X7R/SGC co-localization (Fig. 5E), indicating attenuated SGC activation after EA treatment. As shown in Fig. 5F, there were no obvious difference in the expression of total P38 MAPK protein in the L_4-6_ DRGs among all groups. However, CFA rats exhibited considerably higher p-P38 MAPK protein expression (as measured by the ratio of phosphorylated to total protein) than Con rats. In contrast, the protein expression of p-P38 MAPK in the L_4–6_ DRGs was significantly downregulated in EA-treated rats compared to CFA and CFA + sham EA rats. ELISA results demonstrated that EA markedly inhibited the increase in TNF-α levels induced by CFA modeling (Fig. 5G). To further determine whether EA’s analgesic effect on CFA rats was mediated by peripheral P2X7R, we investigated the effect of the selective P2X7R agonist BzATP on EA’s efficacy. From Day 12 to Day 14 post-CFA injection, BzATP (280 nM/L, 10 μL) was injected intrathecally once per day before EA stimulation (Fig. 6A). BzATP significantly abolished EA's analgesic effects on CFA-induced mechanical allodynia and thermal hyperalgesia in rats (Fig. 6B, C). This antagonistic effect was also observed in naïve rats (Supplementary Fig. 4A, B). Furthermore, it reversed EA-induced reductions in TNF-α levels and p38 MAPK phosphorylation (Fig. 6D, E).

### EA decreased TNF-α secretion from CFA rats’ SGCs via regulating P2X7R

**Fig. 7:**
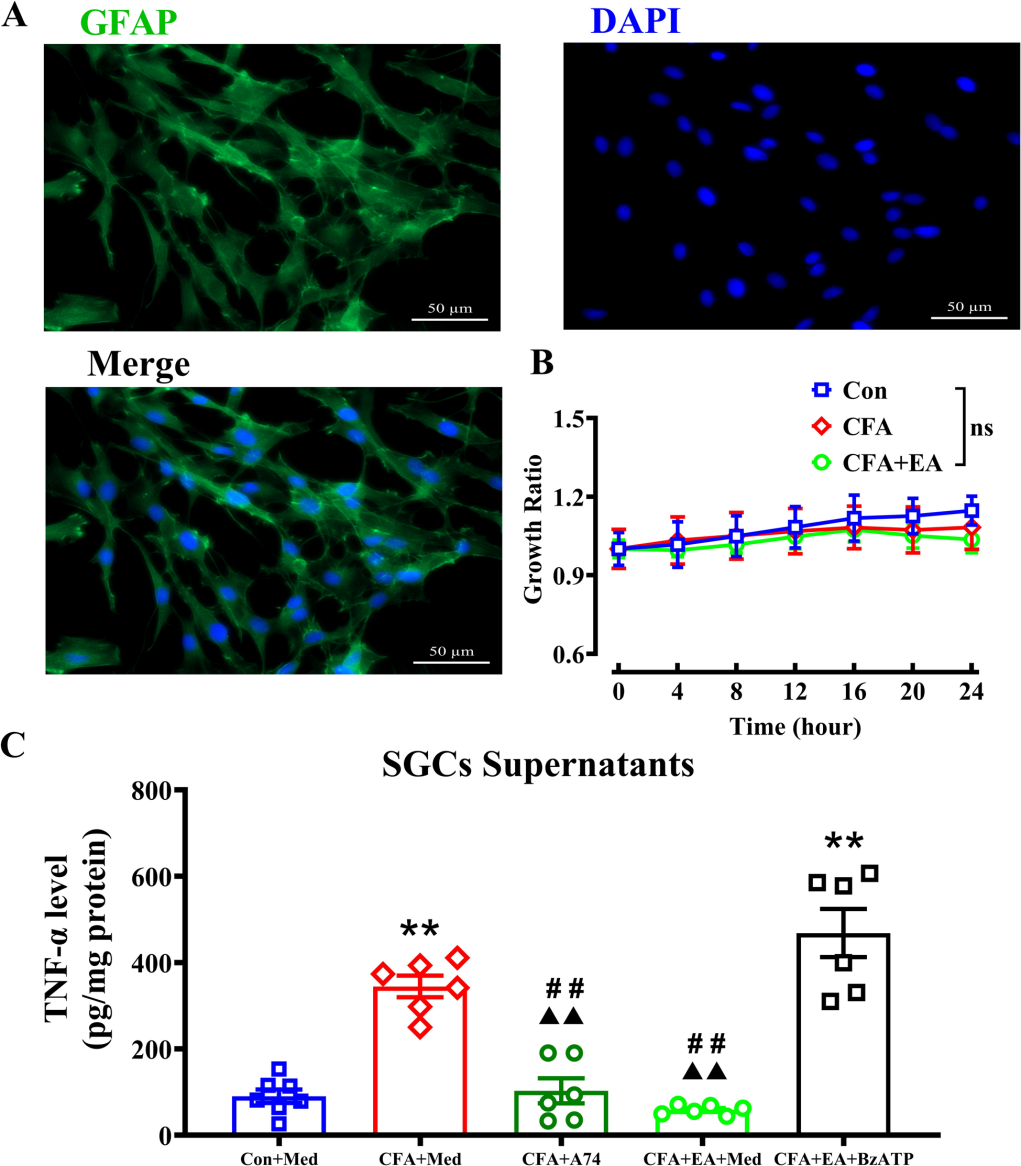
EA decreased TNF-α secretion from CFA rats’ SGCs via regulating P2X7R. **A** Immunostaing showing enriched satellite glial cells cultured successfully in vitro. Green represents the SGCs (marked by GFAP), blue represents the cell nucleus (marked by DAPI). Scale bar indicates 50 μm. **B** There was no difference among the groups in the growth activity of cultured SGCs in vitro. **C** TNF-α concentrations in the supernatants of cultured SGCs in the Con + Medium (Med), CFA + Med, CFA + A740003, CFA + EA + Med and CFA + EA + BzATP groups detected respectively. In the Con + Med group, cultured SGCs in each test had harvested from bilateral L_4-6_ DRGs of one rat and 7 tests in total. In the other groups, cultured SGCs in each test had harvested from ipsilateral L_4-6_ DRGs of two rats and 6 tests per group in total. **p < 0.01 vs. Con + Med group; ##*p* < 0.01 vs. CFA + Med group; (double Filled triangle) *p* < 0.01, vs. CFA + EA + BzATP group. One-way ANOVA followed by Tukey’s post hoc test was used for all comparisons

To further determine the effect of EA on TNF-α secretion from CFA rats’ SGCs and investigate the role of SGCs’ P2X7R in this process, we first cultured SGCs from the L_4-6_ DRGs of rats in each group and then proceeded with pharmacological intervention. The supernatants of the cells in each group were collected, and the concentration of TNF-α was measured to evaluate the changes in TNF-α production secreted by SGCs across groups. Cell specificity was identified using cellular immunofluorescence, where cultured cells were stained and labeled with GFAP, an activation-specific marker for SGCs. The results confirmed successful enrichment of cultured SGCs (Fig. 7A). RTCA results showed that in vitro cultured SGCs had normal growth activity, which was not affected by the addition of the P2X7R-specific antagonist A740003 (final concentration 1 μM) or the agonist BzATP (final concentration 100 μM) (Fig. 7B and S5). As shown in Fig. 7C, SGCs in the CFA + Med group released significantly higher levels of TNF-α compared to the Con + Med group. The P2X7R antagonist 740,003 markedly reduced TNF-α levels in the SGC supernatant compared to the CFA + Med group. Additionally, TNF-α levels in the supernatant were considerably lower in the CFA + EA + Med group compared to the CFA + Med group. When BzATP solution was added to the medium to activate P2X7R in SGCs, the TNF-α levels in the supernatant dramatically increased in the CFA + EA + BzATP group compared to the CFA + EA + Med group. These findings suggest that EA decreases TNF-α secretion from CFA rats’ SGCs, which was related to the activation of SGCs’ P2X7R.

## Discussion

This study explores the role of P2X7R in SGCs and its downstream pathway in chronic inflammatory pain, as well as the cellular mechanisms underlying EA's analgesic effects through the regulation of P2X7R mediated signaling pathway. Our findings indicate that activation of P2X7R in SGCs leads consequently to higher phosphorylation of P38 MAPK, as well as increased secretion of TNF-α from SGCs, contributing to chronic inflammatory pain induced by CFA. Furthermore, EA alleviates CFA-induced inflammatory pain through a cellular mechanism involving the inhibition of P2X7R mediated P38 MAPK/TNF-α pathway. Here, we have presented a novel experimental basis for the EA treatment of inflammatory pain.

As an important communication center between the peripheral and central nervous system, DRG neurons perceive various sensations such as mechanical, thermal, chemical, and noxious stimuli from different parts of the body and transmit sensation information to the cerebral cortex for further processing [51]. DRG neurons do not interact with each other through synaptic contacts, but are surrounded by a layer of SGCs, forming a physically isolated unit [52]. This structural arrangement between neuronal soma and SGCs suggests that their communication is a key determinant of somatic activity [53]. The P2X7R is widely distributed across the central and peripheral nervous systems, as well as the immune system, and plays a significant role in various pain processes [54–57]. Activation of P2X7R within the DRG initiates intra-ganglionic purinergic signaling, triggering neuron-glia interactions and enhancing DRG neuron excitability in vivo [58]. Immunofluorescence labelling in this study confirmed that P2X7R is exclusively located in SGCs surrounding neurons within the DRG of CFA rats, consistent with previous reports [59]. Furthermore, we observed a significant upregulation of P2X7R expression in L_4–6_ DRGs of CFA rats, aligning with earlier findings [21]. Pharmacological inhibition of P2X7R using the specific antagonist A740003 markedly reduced pain hypersensitivity in CFA-treated animals. Similarly, intra-DRG injection of an AAV-vectored shRNA targeting the knockdown of P2X7R expression in SGCs attenuated both mechanical nociceptive abnormalities and thermal hyperalgesia. These findings underscore the critical role of P2X7R in SGCs in mediating pain hypersensitivity associated with chronic inflammatory pain. Acupuncture (including electroacupuncture) is widely recognized as a complementary and alternative therapy for pain relief. However, the role of SGCs’ P2X7R in EA’s analgesic effects on inflammatory pain models has remained unclear. Our findings suggest that EA may modulate P2X7R expression in satellite glial cells (SGCs) through multiple potential pathways: First, EA's well-documented ability to activate specific sensory fiber types [60, 61] could differentially influence SGC networks. Low-frequency EA (2 Hz) preferentially activates Aδ fibers and low-threshold mechanoreceptors (LTMRs) [60, 62], which form functional gap junctions with SGCs. This may trigger activity-dependent downregulation of P2X7Rs through purinergic signaling (ATP release) and subsequent glial network modulation. Second, our observed reduction in p38 phosphorylation (Fig. 2E) suggests EA may interrupt the P2X7R-mediated neuron-glial feedback loop, where: (1) Nociceptor activation releases ATP → (2) Activates P2X7Rs on SGCs → (3) Promotes TNF-α release → (4) Further sensitizes neurons. The transient nature of EA effects (Suppl. Figure 3) supports this signaling-mediated mechanism rather than permanent receptor alteration. However, whether LTMRs versus nociceptors play dominant roles requires further investigation using selective fiber ablation models. Importantly, our rAAV-mediated SGC-specific knockdown (Fig. 3) confirms that P2X7R modulation in SGCs alone is sufficient for significant analgesic effects, though central mechanisms may contribute synergistically. This study advances our understanding of the mechanisms through which EA alleviates inflammatory pain by targeting P2X7R-mediated pathways.

To further elucidate the effect of P2X7R activation in SGCs within chronic inflammatory pain models, we focused on the role and function of SGCs. These cells are known to produce a wide range of biologically active substances, including ATP, glutamate, and cytokines such as tumor necrosis factor, interleukin-1 beta, and fractalkine [63]. Among these, TNF-α is a well-established pro-inflammatory cytokine that functions as a pain mediator and plays a crucial role in the development of peripheral and central sensitization [64]. However, it remains unclear whether P2X7R activation in SGCs of CFA rats can regulate TNF-α production and how EA influences this process. To gain further insight into the changes in TNF-α levels released by SGCs, we cultivated SGCs in primary cultures derived from the L_4-6_ DRGs of rats in the Con, CFA, and CFA + EA groups. Our results demonstrated that SGCs from CFA rats released significantly higher levels of TNF-α, a rise that was attenuated by blocking P2X7R function. Notably, SGCs from the CFA + EA group produced significantly lower levels of TNF-α compared to those from the CFA + Med group. Furthermore, when SGCs were co-cultivated with the P2X7R agonist BzATP, the supernatant of these cells showed a substantially higher level of TNF-α compared to the CFA + EA group. These findings suggest that P2X7R activation contributes to nociceptive sensitization in CFA-induced chronic inflammatory pain by promoting increased TNF-α release from SGCs. Importantly, these effects can be effectively mitigated by EA, highlighting its potential to modulate inflammatory responses at the cellular level.

It is widely recognized that the activation of MAPK pathways, particularly P38 MAPK, alters neural plasticity and contributes to pain hypersensitivity [65]. Significant increases in phosphorylated P38 MAPK have been observed in both acute and persistent inflammatory pain models within spinal cord and DRG tissues [66–69]. The activation of these pathways enhances the expression of inflammatory factors such as TNF-α and IL-1β, which further promote the release of pain mediators, ultimately leading to nociceptor sensitization. Previous studies have shown that phosphorylated P38 MAPK expression is upregulated in L_4-6_ DRGs of mice with CFA-induced inflammatory pain, and that pain hypersensitivity in these models can be alleviated by inhibiting P38 MAPK phosphorylation [34]. Interestingly, blocking P2X7R with the antagonist A740003 has been shown to reduce p-P38 MAPK expression in DRGs [70]. Given the critical role of inflammatory pain in its development and maintenance, we sought to investigate the relationship between P38 MAPK phosphorylation and P2X7R activation in CFA-induced chronic inflammatory pain. Our results demonstrated that P38 MAPK phosphorylation was significantly elevated in the L_4-6_ DRGs of CFA rats at 14 days post-modelling, consistent with previous findings [34]. Notably, inhibition of P2X7R function with A740003, or knockdown of P2X7R expression, significantly suppressed P38 MAPK activation. These findings suggest that P2X7R-mediated P38 MAPK activation plays a pivotal role in the pathophysiology of CFA-induced chronic inflammatory pain.

We therefore speculate that the analgesic effect of EA mediated through P2X7R suppression may primarily involve the inhibition of the P38 MAPK signaling pathway. Pharmacological P2X7R antagonists (A740003) mimic EA’s analgesic effects, but their clinical utility is limited by systemic side effects. In contrast, EA provides localized P2X7R inhibition without off-target toxicity. SGC-specific P2X7R knockdown replicates EA’s benefits, validating SGCs as a cellular target for precision analgesia. Unlike NSAIDs or opioids, EA avoids gastrointestinal, cardiovascular, or addictive risks. Our open-field tests confirmed no motor impairment. A previous study indicated that blocking the activation of the spinal P38 MAPK pathway may represent one of the main central mechanisms underlying the anti-inflammatory pain effects of EA in CFA rats [71, 72]. In this study, EA treatment significantly reduced the expression of P2X7R, p-P38 MAPK, and the secretion of TNF-α from SGCs in L_4-6_ DRGs of CFA rats. These results suggest that EA exerts an analgesic effect on CFA-induced inflammatory pain, potentially through the inhibition of P2X7R-mediated P38 MAPK/TNF-α signaling in SCGs. The cellular mechanism underlying EA's peripheral analgesic effects in CFA-induced inflammatory pain may be explained by our findings.

Our investigation demonstrated that the elevated expression of P2X7R, TNF-α, and P38 MAPK activation in SGCs are all intrinsically connected to the maintenance of chronic inflammatory pain. EA appears to alleviate pain by inhibiting these pathways. However, there is no direct evidence demonstrating the specific impact of suppressing P38 MAPK activation or neutralizing TNF-α expression in SGCs on chronic inflammatory pain, nor the precise interconnections between these mechanisms. Therefore, further research employing advanced and precise experimental methodologies is required to confirm the full role of the P2X7R-mediated P38 MAPK/TNF-α pathway in SGCs in the analgesic effects of EA. Such studies would provide deeper insights into the molecular underpinnings of EA's role in managing inflammatory pain.

## Conclusion

This study demonstrates a precise role of P2X7R in SGCs in mediating peripheral nociceptive sensitization in chronic inflammatory pain. EA exerts its anti-inflammatory and analgesic effects by inhibiting P2X7R expression in SGCs, thereby suppressing activation of the P38 MAPK/TNF-α pathway. These findings highlight the potential of targeting peripheral P2X7R in SGCs to elucidate the cellular mechanisms underlying EA’s analgesic effects on inflammatory pain.

## Supplementary Information

Supplemental Figure 1. Effects of P2X7 receptor antagonists A740003 on pain behaviors in naïve rats. **A** Effect of P2X7R antagonist A740003 (250 nmol/L in 10 μL) on mechanical allodynia in naïve rats, n = 5/group. **B** Effect of A740003 on thermal hyperalgesia in naïve rats, n = 5/group. *p < 0.05, **p < 0.01 vs. Con + Veh group. Two-way ANOVA with repeated measures followed by Tukey’s post hoc test was used for comparisons in A-B.

Supplemental Figure 2. Effects of 7-session electroacupuncture on motor function in rats. **A** Diagram showing the experiments’ design. **B** Total distance of the OFT, n = 10/group. **C** The representative trajectory diagram of the OFT. Student’s t test was used for comparisons in B.

Supplemental Figure 3. The analgesic effects of EA were no longer detectable following treatment cessation in CFA rats. **A** Time course showing the effect of EA (D8-D14) treatment on mechanical allodynia in CFA rats. n = 6/group. **B** Time course showing the effect of EA (D8-D14) treatment on thermal hyperalgesia in CFA rats. n = 6/group. *p < 0.05, **p < 0.01, vs. Con group; #*p* < 0.05, ##*p* < 0.01 vs. CFA group; ▲*p* < 0.05, ▲▲*p* < 0.01, vs. CFA + sham EA group. Two-way ANOVA with repeated measures followed by Tukey’s post hoc test was used for comparisons in A-B.

Supplemental Figure 4. Effects of P2X7 receptor agonist BzATP on pain behaviors in naïve rats. **A** Effect of P2X7R agonist BzATP (280 nmol/L in 10 μL) on mechanical allodynia in naïve rats, n = 5/group. **B** Effect of BzATP on thermal hyperalgesia in naïve rats, n = 5/group. *p < 0.05, **p < 0.01 vs. Con + Veh group. Two-way ANOVA with repeated measures followed by Tukey’s post hoc test was used for comparisons in A-B.

Supplemental Figure 5. The effect of different interventions on the growth activity of SGCs cultured in vitro. **A** Growth activity of SGCs in vitro had not influenced during 24 h before or after co-cultured with A740003 (1 μM). **B** Growth ratio curve showing the growth activity of SGCs in vitro has not inhibited during 24 h before or after co-cultured with BzATP (100 μM). Two-way ANOVA with repeated measures followed by Tukey’s post hoc test was used for all comparisons.

## Data Availability

The key data are contained in the figures. The datasets used and/or analyzed during this study can be obtained from the corresponding author Dr. Yi Liang on reasonable requests.

## Abbreviations

NSAIDs: Non-steroidal anti-inflammatory drugs
DRG: Dorsal root ganglia
SGCs: Satellite glial cells
P2X7R: P2X7 purinergic receptor
NMDAR: N-methyl-D-aspartate receptor
GLAST: Glutamate-aspartate transporter
EA: Electroacupuncture
CFA: Complete Freund’s adjuvant
NS: Normal saline
PWLs: Paw withdraw latencies
OFT: Open field testing
RTCA: Real-time cell proliferation analysis
ELISA: Enzyme-linked immunosorbent assay
PWTs: 50% Paw withdraw thresholds
AUC: Area under the curve
Con: Control
BL: Baseline
i.pl.: Intraplantar
i.t.: Intrathecal injection
A740003: P2X7R-specific antagonist A740003
p-P38: Phosphorylated protein of P38 MAPK (Thr180/Tyr182)
t-P38: Total protein of P38 MAPK
rAAV: Recombination adeno-associated virus
Veh: Vehicle.
ns: No significant
