# Supplementary figures and images for: Inhibition of P2X7 receptor in satellite glial cells contributes to electroacupuncture's analgesia in rats with CFA-induced inflammatory pain
Source: Chin Med. 2025 Oct 5;20:159. doi: 10.1186/s13020-025-01227-6 (PMC12497349; doi:10.1186/s13020-025-01227-6)

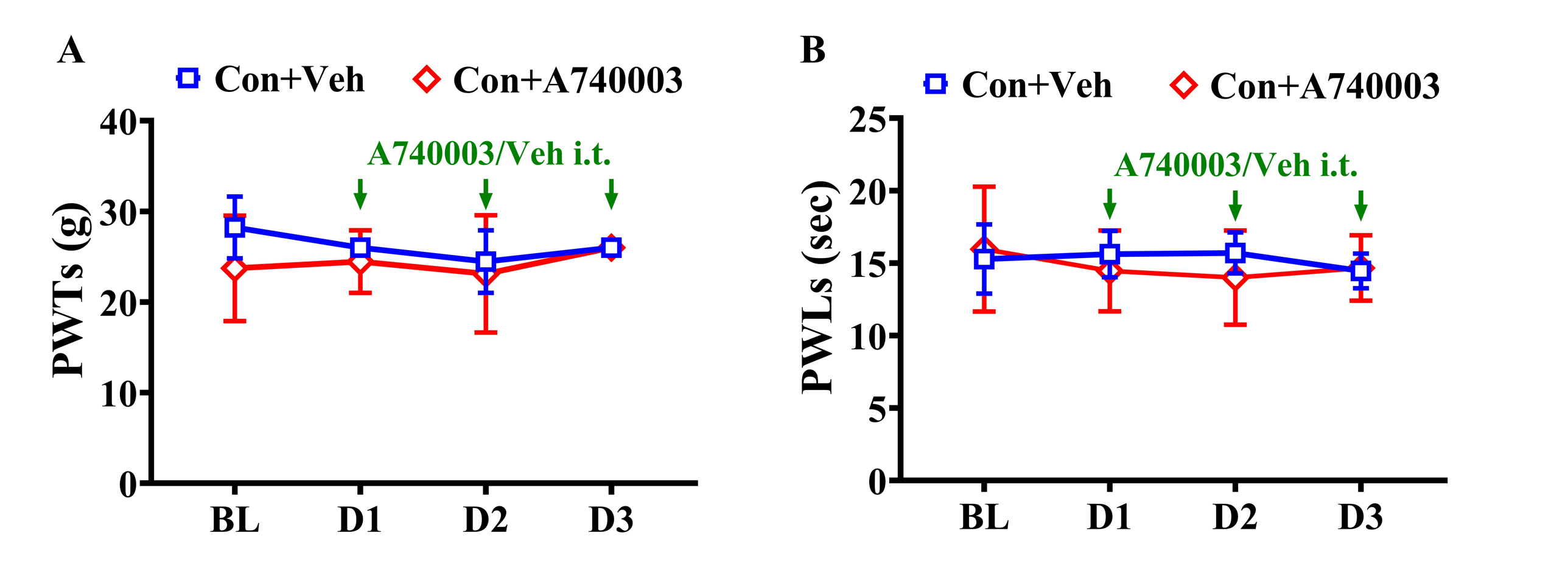

Supplement: Supplementary file 1 — Supplemental Figure 1. Effects of P2X7 receptor antagonists A740003 on pain behaviors in naïve rats. A Effect of P2X7R antagonist A740003 (250 nmol/L in 10 μL) on mechanical allodynia in naïve rats, n = 5/group. B Effect of A740003 on thermal hyperalgesia in naïve rats, n = 5/group. *p < 0.05, **p < 0.01 vs. Con + Veh group. Two-way ANOVA with repeated measures followed by Tukey’s post hoc test was used for comparisons in A-B. [file 13020_2025_1227_MOESM1_ESM.jpg]

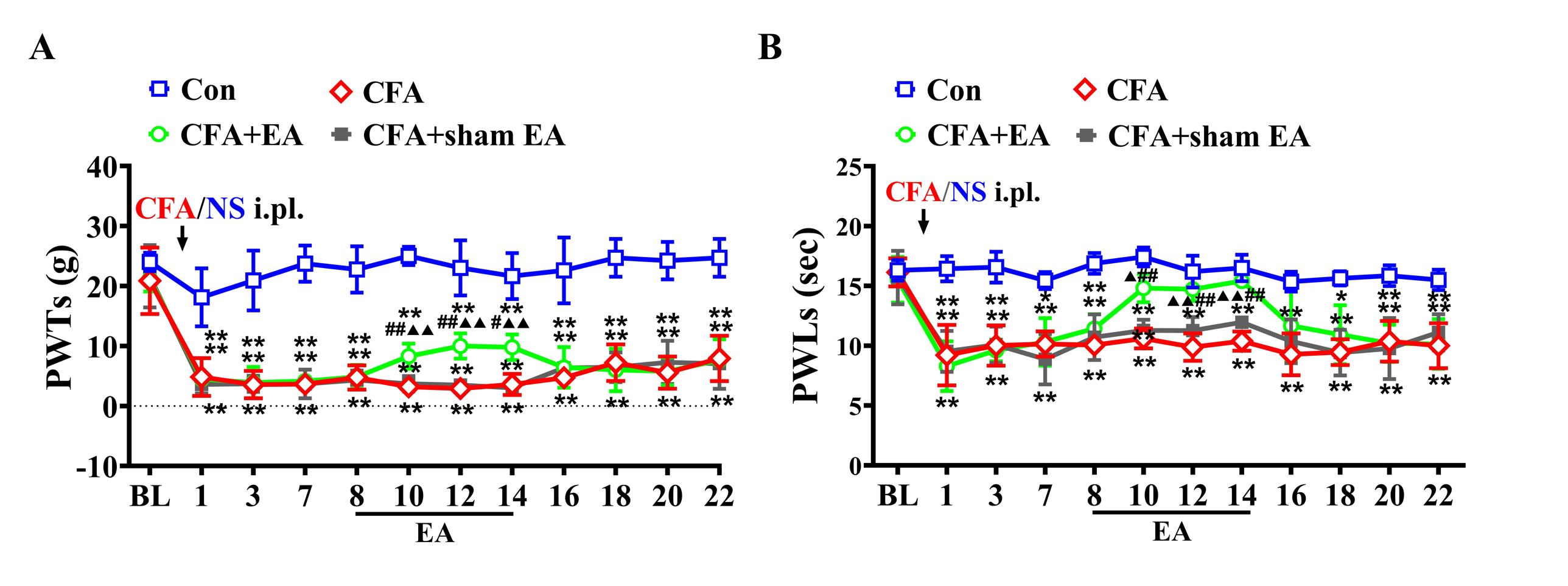

Supplement: Supplementary file 3 — Supplemental Figure 3. The analgesic effects of EA were no longer detectable following treatment cessation in CFA rats. A Time course showing the effect of EA (D8-D14) treatment on mechanical allodynia in CFA rats. n = 6/group. B Time course showing the effect of EA (D8-D14) treatment on thermal hyperalgesia in CFA rats. n = 6/group. *p < 0.05, **p < 0.01, vs. Con group; #p < 0.05, ##p < 0.01 vs. CFA group; ▲p < 0.05, ▲▲p < 0.01, vs. CFA + sham EA group. Two-way ANOVA with repeated measures followed by Tukey’s post hoc test was used for comparisons in A-B. [file 13020_2025_1227_MOESM3_ESM.jpg]

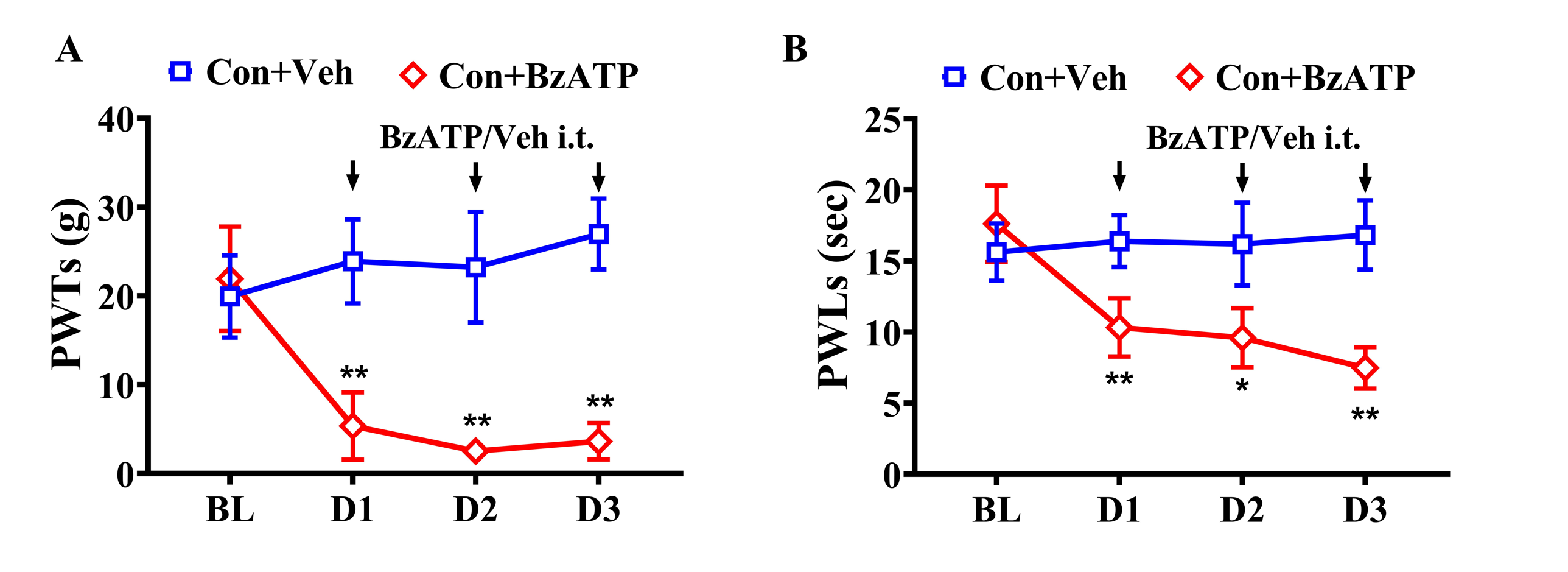

Supplement: Supplementary file 4 — Supplemental Figure 4. Effects of P2X7 receptor agonist BzATP on pain behaviors in naïve rats. A Effect of P2X7R agonist BzATP (280 nmol/L in 10 μL) on mechanical allodynia in naïve rats, n = 5/group. B Effect of BzATP on thermal hyperalgesia in naïve rats, n = 5/group. *p < 0.05, **p < 0.01 vs. Con + Veh group. Two-way ANOVA with repeated measures followed by Tukey’s post hoc test was used for comparisons in A-B. [file 13020_2025_1227_MOESM4_ESM.jpg]
